# A User Authentication Scheme Using Physiological and Behavioral Biometrics for Multitouch Devices

**DOI:** 10.1155/2014/781234

**Published:** 2014-07-24

**Authors:** Chorng-Shiuh Koong, Tzu-I Yang, Chien-Chao Tseng

**Affiliations:** ^1^Department of Computer Science, National Taichung University of Education, Taichung 40306, Taiwan; ^2^Department of Computer Science, National Chiao Tung University, Hsinchu 30010, Taiwan

## Abstract

With the rapid growth of mobile network, tablets and smart phones have become sorts of keys to access personal secured services in our daily life. People use these devices to manage personal finances, shop on the Internet, and even pay at vending machines. Besides, it also helps us get connected with friends and business partners through social network applications, which were widely used as personal identifications in both real and virtual societies. However, these devices use inherently weak authentication mechanism, based upon passwords and PINs that is not changed all the time. Although forcing users to change password periodically can enhance the security level, it may also be considered annoyances for users. Biometric technologies are straightforward because of the simple authentication process. However, most of the traditional biometrics methodologies require diverse equipment to acquire biometric information, which may be expensive and not portable. This paper proposes a multibiometric user authentication scheme with both physiological and behavioral biometrics. Only simple rotations with fingers on multitouch devices are required to enhance the security level without annoyances for users. In addition, the user credential is replaceable to prevent from the privacy leakage.

## 1. Introduction

Owing to the rapid growth of mobile device computation power, personal digital assistants, smart phones, and tablets have become sort of keys controlling our daily life. Most of them provide user friendly interfaces that can be easily operated through fingers and multitouch display. Mobile devices are not only used to make calls, receive messages, take photos, and play games, but also give all kinds of help for both personal business and financial services. Users can transfer money, manage bank accounts, pay for products and game credits using digital money, sell stocks, and even pay vending machines using mobile devices online almost anytime, anywhere. As a consequence, user authentication for mobile devices has become an important issue [1]. User authentication is the act of confirming a person using personal identities, which often involves verifying at least one form of identification. There are three major factors to authenticate users, based on something the user knows (password and challenge response), something the user has (ID, security token, device, and equipment), and something the user is (fingerprint, DNA, and other biometric identifiers). Each authentication factor covers a range of elements used to authenticate a person's identity, which can be used to grant the access authorization, approve a transaction request, and sign documents.

The authentication on mobile devices can currently be classified into three major approaches. PIN (personal identification number) or passwords, the secret-knowledge approach, are the most popular authentications with the features of quick operation and low cost. Financial PINs are often four-digit numbers in the range 0000–9999, resulting in 10,000 possible numbers. However, some banks do not give out numbers where all digits are identical, consecutive, numbers that start with one or more zeroes, or the last four digits of your social security number. Although a more complicated approach named two-factor authentication [2] uses SMS combined with the one time password (OTP) user authentication scheme that is widely used by leading commercial companies, it still may suffer from the phishing attacks [3]. On the other hand, passwords seem alternatively more secure because of the more possible combinations by using all symbols and alphabets. Unfortunately, people always use the same password everywhere and rarely change it. Although the security level can be enhanced through forcing users to change password periodically, it may also add annoyances for users. On the other hand, sharing passwords and phishing attacks are serious problems that happen frequently in our daily life [3, 4]. Phishing attack is the act of attempting to steal sensitive information, such as passwords and credit card details (knowledge factor), by masquerading as a trustworthy entity in an electronic communication [4]. Phishing attack is typically performed through email spoofing [5], instant messaging [6], and SMS services [7]. It often leads users to enter personal information on a fraudulent website, which makes the user look and feel the same as the legitimate one. Although several antiphishing technologies were revealed against these malicious behaviors, it still needs user training and public awareness to make it work.

The second approach is the SIM (subscriber identification module), so-called token-based system, which is an integrated circuit that securely stores the IMSI (international mobile subscriber identity) and the related keys used to identify and authenticate subscribers on mobile telephony devices. A SIM is embedded into a removable SIM card, which can be transferred between different mobile devices. It is usually used for small payments, such as vending machines and game point cards. However, removing the SIM is not recommended because it would cause the loss of signal and other inconvenient manners.

**Figure 1:**
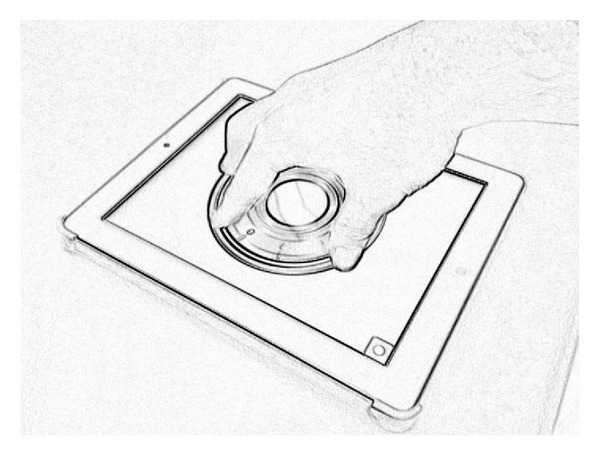
An example of pbLogon.

The last one is authentication through biometric characteristics, which are unique enough to distinguish each person. The development of the biometric authentication technology has the trend of replacing the traditional verification method and can solve the traditional security problems. Biometric approaches are typically divided into two categories: physiological and behavioral biometrics. Physiologic biometrics refer to physical measurements of the human body, including face, fingerprint, hand geometry, retina, and iris (please refer to Figure 1). The recognition system based on physiological characteristics has a relatively high accuracy [8, 9]. However, the fingerprint of those people working in chemical industries is often affected. On the other hand, people affected with diabetes, the eyes also get affected resulting in differences. In addition, the use of physiologic biometrics introduces privacy concern since the body characteristics are irreplaceable [10].

Behavioral biometrics relate to a specific behavior of a human while performing some tasks, such as handwriting, speaking, and typing [11, 12]. Usually, handwriting recognition used signature as identity, which means it is not suitable for general-purpose authentications [13]. Voice biometric authentication uses the voice pattern to verify the identity of the individual. However, automatic speaker authentication systems may be affected by the extreme emotional states, sickness, and aging of the speaker and noise [14]. Keystroke dynamics [15] is considered of the most successful behavioral biometrics with the benefit of almost free as the only hardware required is the physical keyboard. Users' keystroke rhythms are measured to develop a unique biometric template for future authentication. However, a person's hands can also get tired or sweated after prolonged periods of typing which resulted in major pattern differences [16]. In addition, typing patterns may vary based on the keyboard layout, the person's posture and language dependency. On the whole, both physiological and behavioral biometrics approaches require different equipment for extracting the characteristics for verification, which may not be portable and can be expensive.

This study proposes a novel scheme pbLogon (physiological and behavioral user authentications), which combines both physiological and behavioral biometric characteristics. The multitouch panel is the only equipment required, which is built in almost every modern mobile device. It aimed to provide a strong user authentication environment, which uses at least two authentication factors, also suggested and grounded by different research [1, 17].

## 2. Related Works

### 2.1. Physiological Biometrics

Physiology is the characteristic of the body and thus it varies from person to person, including fingerprint, hand geometry, face, and iris and retina recognition. The fingerprint [18] is using patterns which are aggregate characteristics of ridges and minutia points. It provides an over 99% recognition accuracy that is widely used by governments and leading industries [19]. The palm print technology [20] can be considered the same despite of the scale size being different. A face recognition technique [21] is applications that identify or verify a person automatically from a digital image or a video frame from a video source. It is the most natural mean of biometric identification. Facial metric technology relies on the manufacture of the specific face recognition feature, such as the position of eyes, nose and mouth, and distances between these features. Face recognition may suffer from the rise of wrong identifications owing to the surrounding environment and lighting affecting the quality of images acquired [22]. As for the iris technology [23], it uses the colored area that surrounds the pupil. Iris patterns are unique, which can be a combination of specific characteristics known as the corona, crypts, filaments, freckles, pits, furrows, striations, and rings. As for retina geometry technology [24], it is based on the pattern of blood vessel in the retina that has unique patterns from person to person. Hand geometry technology [25] is based on the fact that nearly every person's hand is shaped differently and that the shape of human hands does not change after a certain age. These techniques include the estimation of length, width, thickness, and surface area of the hand. Essentially, hand identification approaches can be classified into two categories based upon the nature of image acquisition: contact-based and contact-free. With the contact-based approach, users are often asked to place their hands on a flat surface or a digital scanner. Recently, the contact-free approaches are increasingly being considered because of their characteristics in user acceptability, hand distortion avoidance, and hygienic concerns [26]. Besides, more information can be obtained since contact-free approaches can obtain both 2D and 3D hand geometry information [27]. Both of them need extra equipment, which is not portable. In our proposed scheme, we introduce a novel hand geometry technique, which is the contact-based but measures the relative position of each finger instead, since the researchers [8, 25, 26, 28] stand guarantee for hand geometry. In addition, more unique features such as the natural pose, rotation angle, and polygon area of different fingers, which come by using the touch panel in advance.

### 2.2. Behavioral Biometrics

Behavioral biometrics are the behavioral characteristics that related to the pattern of people doing something, such as signature, typing rhythm [29], gait [11], and mouse movement [30]. The signature recognition is based on the dynamics of making the signature, rather than a direct comparison of the signature itself afterwards. The dynamic is measured as a mean of the pressure, direction, acceleration and the length of the strikes, and dynamic number of strokes and their duration. A keystroke dynamic is based on the assumption that different people have unique habitual rhythm patterns in the ways they typed and analyzed using statistical technique traditionally. By introducing the pattern recognition technique, such as *z*-test, Bayesian classifiers, and neural network, they have brought the recognition rate to a new level [15, 29]. Hence, the analysis of keystroke becomes one of the most useful authentication schemes because it is based on the user's experience and individual skills. However, people who use different input methods such as phonetic may suffer from the language problem which also causes the embarrassments of detecting biometrics. Recently, a few studies have considered the keystroke dynamics of mobile devices, which have investigated the keystroke recognition using the virtual keyboard [31]. However, Clarke and Furnell [32] investigated the feasibility of authenticating users based on their typing habits using the neural network showing that only partial participants' characteristics can be discriminated. Furthermore, most of the biometric approaches require additional equipment to verify these biometric characteristics, which may also increase the manufacturing cost.

### 2.3. Gesture-Based User Identification

Gesture-based user identification uses human body gestures and gaits to recognize the user. Researchers use different equipment, such as accelerometer [33], video [34], and Kinect [35], to track the patterns while human walking or performing poses in different ways. The benefits can be the easy operation while performing user authentication. However, extra equipment may be required to perform user login, which means it may not be portable and not suitable for daily use.

Sae-Bae et al. [36] advocated that using only hand gestures acquired by multitouch panels can reach a 90% accuracy rate with a single gesture. They use the hand operational features, which include parallel, closed, opened, and circular hand movements. However, how to normalize these operations can be relatively difficult and hard to detect if the hand moving area can be varied case by case while using different Apps. Therefore, this study introduces pbLogon, which provided the carrier, virtual wheel lock, to limit the operation area of users and further increase the usability and raise the accuracy while acquiring users' biometric information. Besides, more analysis can be carried out through recording the operational force that is provided by the built-in gyroscope. In addition, resizing the virtual wheel lock can bring the benefit of revocable biometric templates.

### 2.4. Biometric Privacy Concerns

One disadvantage of biometrics is that they cannot be easily revoked. Physiological biometrics is generally irreplaceable which means it may suffer from the privacy issue [37, 38]. Although some research provides a more advanced protection to prevent from the privacy leakage of user template, it may also increase the complexity and power consumption of mobile devices [39, 40].

Another serious problem is the irreplaceable of biometric characteristics. Traditionally, while the user account has been compromised, the passive way is to ask the user to change password, and the more active way is to change the layout of keyboards that prevent from further remote stolen. However, the extraction of new biometrics can be limited because of the quantity limitation, such as ten fingerprints. As a consequence, it is also important to provide both revocable and replaceable biometric authentication schemes. This study proposes a novel biometric authentication scheme, which includes the features of both physiological and behavioral biometrics so-called pbLogon to solve the problems mentioned above. It aims to build a multifactor user authentication system, which is a strong user authentication, biometric-based, and replaceable as a privacy concern.

## 3. pbLogon Scheme

Figure 1 brings the example of pbLogon, which uses an Apple iPad2 as the equipment for gathering personal biometrics. The iPad2 has a multitouch panel, which can track up to 5 fingers simultaneously. The pbLogon system will only react while user performs rotations on the virtual wheel lock area. Users can input their credentials by rotating the wheels either clockwise or counterclockwise. Then both physiological and behavioral biometrics can be obtained through these operations.

### 3.1. Extraction of Physiological and Behavioral Biometrics

**Figure 2:**
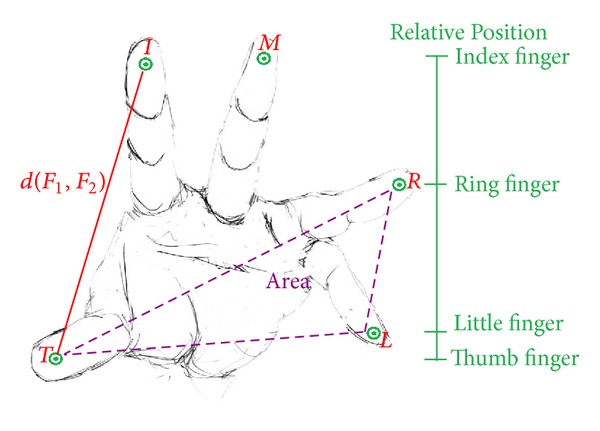
Example of hand's physiological biometric.

It is relatively easy to gather users' physiological biometric information using touch panels rather than traditional optical devices. The physiological phase extracts the finger information, which may include the relative position, distance of different fingertips, and area of each three fingers (please refer to Figure 2). To correctly compare any two biometric templates, they need to be acquired and stored in a consistent order.

Hence the first step is to reorder the touch sequences into a canonical form. The standard order employed was the touches generated by thumb, index, middle, ring, and little fingers. It is hard to determine the right sequences because the acquisition process may capture points in an arbitrary order depending on which fingertips made contact with the touch panel first. To correctly match touch sequences with fingers we use known natural characteristics of human hand geometry. First, we sort the acquired data with *x*-axis in ascending order. Then we check the *y*-axis for the thumb, which is located at the lowest position in a natural pose. The corresponding order then can be determined by comparing the thumb to index and little fingers. More detailed information will be provided in Algorithm section. By examining the information the user provided, it can easily collect the user's sketch and the composition of his fingers. In addition, whether index finger is longer or the ring finger is decided by personal DNAs which can also be used to identify the user.

**Figure 3:**
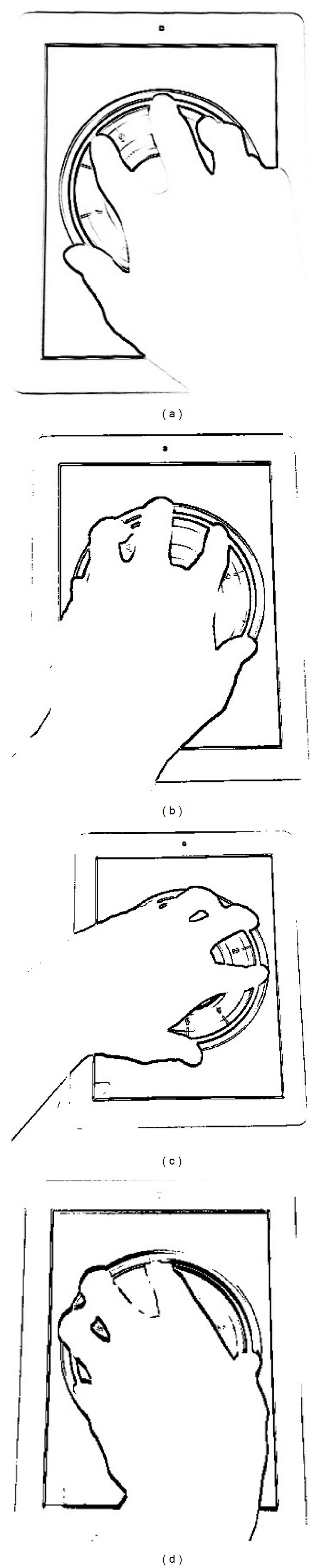
An example of behavioral biometrics. (a) Presents a right-handed user. (b) Presents a left-handed user. (c) Presents a left-handed user with clockwise rotation. (d) Presents a left-handed used with counterclockwise rotation.

In behavioral phases, analyzing the rotational dynamics can reveal more behavioral information.  Figure 3(b) brings the example of a left-handed user with pbLogon operations. A left-handed user can rotate more in the clockwise direction rather than the counterclockwise (Figure 3(c)). On the other hand, a left-handed user may also rotate much faster for clockwise than counterclockwise direction. In addition, tracking the moving speed and path may also help identify the user. Both phases provide rich information to decide whether the user with corresponding ID and password is the compromised one or not. On the other hand, a virtual wheel lock is adapted to limit the operational area while using pbLogon. The main purpose is to help the biometric extraction engine for gathering biometric characteristics more accurately. If the user touches outside of the virtual wheel lock, then pbLogon will not start the extraction process and will prompt the user to put fingers on the wheel lock to input passwords.

In our proposed system, the identity of the user is given to the system along with a proof of the biometric; that is, only the biometric information is used for user authentication. Correctness of the identity is then evaluated by the system; passwords are not involved in the authentication process. After that, either accepting or rejecting the user is given based on the evaluation result. In order to verify the proof, the system needs to have a prior knowledge, for example, the user profile. Generally, there exist two stages in a user authentication system: enrollment and verification stages. The purpose of enrollment stage is to register the users' data in the system by acquiring, extracting and storing biometric templates corresponding to the user. In the verification stage, the input biometric instance is compared with the stored biometric templates for the claimed identity in order to authenticate a user.

### 3.2. Notation

The notations used throughout this paper are listed as Notations section.* Password* is composed of *n*-digits numeric password, for example, PIN, which is given by rotating the wheel lock displayed on screen. *N* is the total number of fingers that you put on the touch screen. *G* is the function calculating the number of corresponding fingers *F*_*i*_ acquired by touch panel, which means *N* = *G*(*F*_*i*_). It also detects the hand pose. *D* is the set of the relative distance of each two fingers, for example, *D* = {*d*_*i*,*j*_∣*d*_*i*,*j*_ = *d*(*F*_*i*_, *F*_*j*_), ∀0 < *i* ≠ *j* < 6}. *A* is the set of areas that formed by any three fingers, for example, *A* = {*a*_*i*,*j*,*k*_∣*a*_*i*,*j*,*k*_ = area(*F*_*i*_, *F*_*j*_, *F*_*k*_), ∀0 < *i* ≠ *j* ≠ *k* < 6}. *L* indicates the relative length of index and ring fingers; if an index finger is longer than ring fingers, *L* will be one, and otherwise it will be zero. *R* records whether the user is left-handed or right-handed. *V* is the set of velocity of the user performing wheel lock for digits, which also contain the information about the rotational direction, for example, record the counterclockwise rotations with negative values.

### 3.3. Assumptions

There are a few assumptions to proceed with the experiments and the usage of pbLogon. First the user is willing and wants to log into the system. Second, only natural hand poses and normal operations are accepted. Natural hand poses can be detected through the horizontal angle formed by the thumb and little fingers. Third, pbLogon only accepts and verifies the input by handheld poses. The main reason is that with handheld poses, more behavioral biometric information can be obtained through accelerometer and gyroscope. Fourth, pbLogon only allows the hand, the user registered, to login pbLogon. Uncooperated operations are prohibited and considered misuses and attacks.

### 3.4. Algorithms

Several algorithms were proposed to restore the hand pose and extract the physiological and behavioral biometrics.

#### 3.4.1. Tablet Orientation Detection

**Figure 4:**
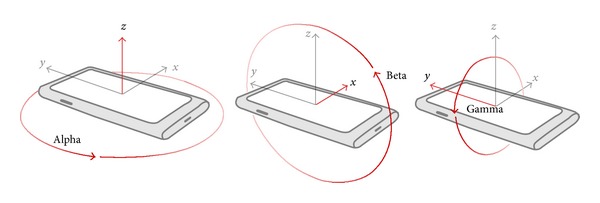
The device orientation definition.

In order to obtain the dynamic orientation while user performing the virtual wheel lock, the tablet orientation detection is required to check if the user inputs their credential by holding the tablet. The gyroscope is a modern piece of equipment that can report the device orientation and is widely built in most of the handset devices. The DeviceOrientationEvent provided by HTML5 and JavaScript is adapted to obtain the orientation information of users' tablet (Algorithm 1 and Figure 4). pbLogon will check the beta factor, which can be used to detect whether the user holds the tablet or puts it on the table.

#### 3.4.2. Hand Natural Poses Restoration

**Figure 5:**
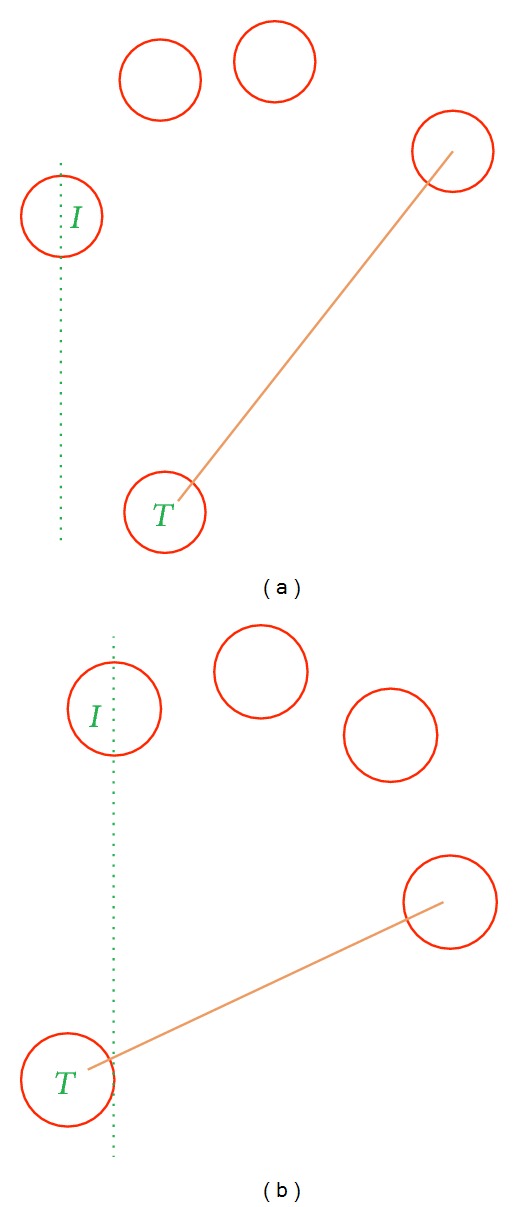
(a) Natural pose with the fingers in order, (b) natural pose with different thumb position, and natural pose with left-handed side user.

Hand nature poses are defined with fingers in the sequence of thumb, index, middle, ring, and little finger. During the experiment, several different poses were shown in Figure 5.  Figure 5(a) demonstrates the idea of natural hand pose in the sequence of thumb, index, middle, ring, and little finger. However, for the experimental participants of this dissertation, most of them operate pbLogon using Figure 5(b) pose, that is, with the sequence of index, thumb, middle, ring, and little finger. Therefore, we proposed the hand pose restoration algorithm to handle different types of hand acquired (Algorithm 2).

#### 3.4.3. Check the User Is Left-Handed or Right-Handed

**Figure 6:**
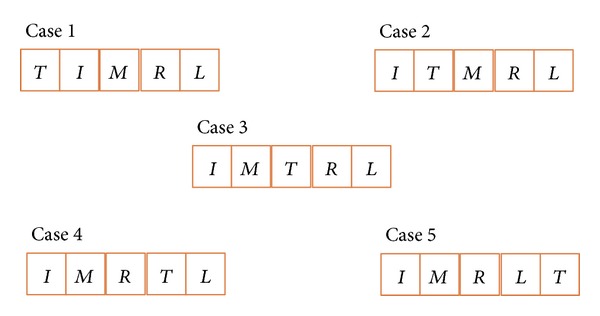
The combinations of thumb in different positions.

Since it is required to calculate the relative distance of each two fingers, the left-handed or right-handed user must be separated to obtain the correct finger orders.  Algorithm 3 gives the check right-hand algorithm. It starts with the sorting of *F*_*i*_ by *x*_*i*_ in ascending order. Then it is required to detect whether the thumb is in ideal position by finding max⁡⁡(*y*_*i*_). Let *A* be the array of *F*_*i*_; if the leftmost of the sorted *A* is the thumb, then it is the ideal natural hand pose. Otherwise, it is required to shift the thumb to leftmost or rightmost for checking hand-side. There will be five cases with the thumb in different positions; please refer to Figure 6. After determining the fingers relative position, the relative distances of thumb to index and little finger are used to estimate the hand-side. It will be a little bit longer while comparing the distance of thumb to little finger than thumb to index finger if the user poses his hand in natural hand pose. Participants of this dissertation all tally with the phenomenon, and it stands until the hand operates over 90 degrees. By comparing the sorted *A*, if thumb to leftmost finger is longer than thumb to rightmost finger, then the system will return 0, which means that the input *F*_*i*_ is a left-handed user. Otherwise, it will return 1, which means that the input *F*_*i*_ is a right-handed user.

#### 3.4.4. Build the User Profile

When MU wants to use *P*, MU must perform the enrollment for building *U*_*i*_. At the enrollment stage, our system starts with checking the number of MU's fingers.

**Step 1 (MU → *P* : *F*_*i*_).**

Then *G*(·) will compute the *F*_*i*_ and return *N* whether MU poses hand in natural or not. It is important that more fingers will bring higher reliability and sturdy biometric information. During the enrollment stage, the natural position of the user's hand is also another important issue. By analyzing the relative position of the thumb and little finger, we can identify whether the user's hand poses in nature or not. It is natural and comfortable if you put your hands touch panels with little finger higher than the thumb in horizontal (please refer to Figures 3(a) and 3(b)). If both requirements are met, pbLogon will start to extract the physiological biometrics including *D*, *A*, *L*, and *R* and then ask MU start to input pass as demands.

**Step 2 (*G*(*F*_*i*_) → *U*_*i*_ = {*D*, *A*, *I*, *R*}).**

*D* of each two fingers can be calculated through the Euclidean distance formula as follows:

(1) $$\begin{array}{c} D=\left\{d_{i,j}\;|\;d_{i,j}=d\left(F_{i},F_{j}\right),\forall 0<i\neq j<6\right\},\\ d\left(F_{i},F_{j}\right)=\sqrt{{\left(X_{i}-X_{j}\right)}^{2}+{\left(Y_{i}-Y_{j}\right)}^{2}}. \end{array}$$

*A* of each three fingers can be calculated through the area formula as follows:

(2) A={Ai,j,k ∣ Ai,j,k=area(Fi,Fj,Fk),∀1≤i≠j≤5}S=d(Fi,Fj)+d(Fj,Fk)+d(Fk,Fi)2a(Fi,Fj,Fk) =S(S−d(Fi,Fj))(S−d(Fj,Fk))(S−d(Fk,Fi)).

For each time MU enters a single digit, *P* will obtain the behavioral biometrics and calculate the *U*_*i*_ of MU.

**Step 3 (MU → *P* : *n*-digit password, *F*_*i*_).**

Consider the following:

(3) $$\begin{array}{c} G\left(F_{i}\right)⟶U_{i}\cup \left\{V,B\right\}. \end{array}$$

By rotating all the *n-*digits into pbLogon, *U*_*i*_ will be established with the procedure Steps 1 to 3 repeatedly. And finally we will have *U*_*i*_ = {*F*_*i*_, *D*, *A*, *L*, *R*, *V*, *B*}.

**Figure 7:**
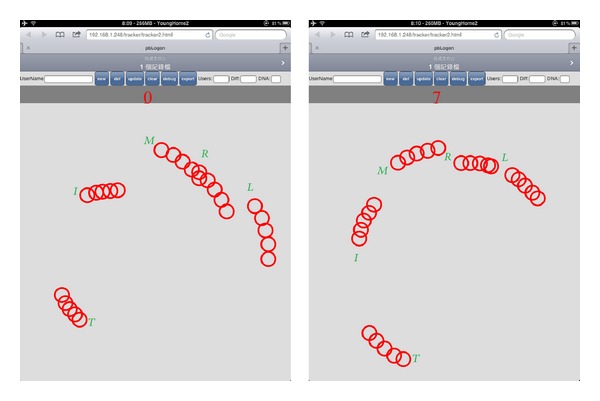
The rotational dynamics.

During the user entering* password*, we can explore even more behavioral biometrics, such as the user is right-handed or left-handed and the rotational dynamics (please refer to Figure 7). A left-handed user can rotate more angles in the direction of counterclockwise. The velocity and rotation dynamics can easily be analyzed from; one may prefer to enter his entire* password* by using the same direction by fingers leaving *P* while the others may use bidirection to finish the job. We believed that there exist more patterns that we can analyze in the future works.

### 3.5. Dissimilarity Score

The decision of users' biometrics depends on the similarity of the input biometrics provided by MU and the stored template *U*_*i*_. In other words, if the dissimilarity score of the input biometric compared to the template is lower than predefined thresholds, the input biometric is verified. Otherwise, the system will reject the user. To calculate the dissimilarity score Δ*S* between the registered user's templates and the input, all distances between the coming gesture and templates are used to calculate the dissimilarity score along with the distances between all the stored templates themselves. For each feature FEA_*i*_, there will be low-bound LB_*i*1_ and up-bound UB_*ik*_ to determine how similar the user is, and the dissimilarity score is calculated by

(4) ΔS=∑|Di,j′−Di,j|, ∀0<i≠j<6Di,j=(Hi,Ck,FEAi)={Hic1〈LBi1,UBik〉⋮⋮ck〈LBik,UBik〉,for 0<i≠j<6. 

## 4. Experiments and Discussions

### 4.1. Experiment

**Figure 8:**
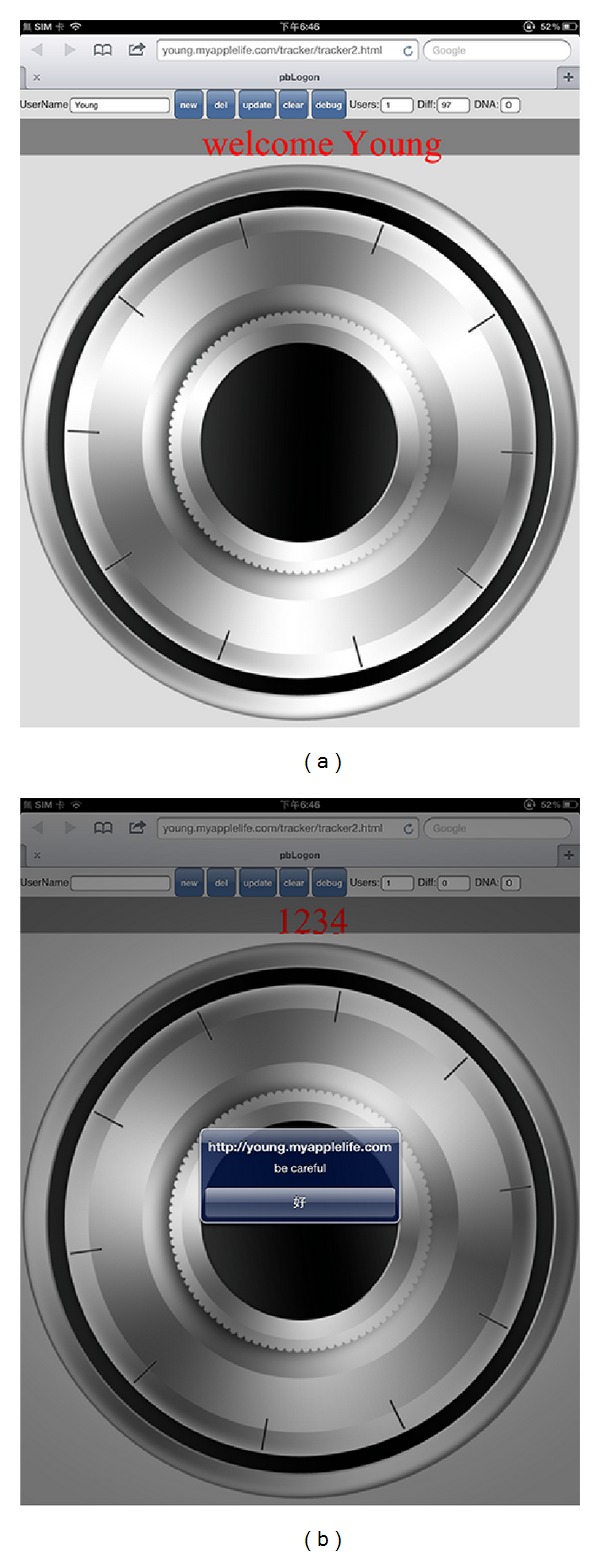
Example of pbAuth. (a) Presents the user logon successfully with a welcome message. (b) Presents the user does not make it due to the threshold we made.

Forty-three participants are involved in the experiment; they are university students from of centeral Taiwan. An HTML5 web application is developed for experiment; iPad2 is adapted which has the ability to track up to 5 fingers simultaneously. As a visualization aid, the application provides simple visual traces of the user's rotations and fingertip movements (Figures 7 and 8). In each session, we first ask the user to input their student ID by rotating the wheel lock in Figure 8 twice. The experiment lasted for one month, and every participant was asked to input ten times (20 records in total).

### 4.2. Analysis of Biometric Data

**Figure 9:**
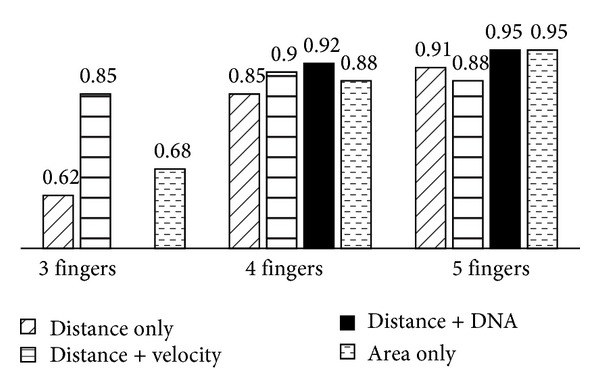
The percentage of successful login rate.

Figure 9 brings the experimental results. If more than four fingers were adopted, most of the classify scheme can reach a near 90% success rate. One of the reasons may be the control difficulties of using only three fingers that lead to the overvariation of fingers' distance and areas. Therefore, it is suggested to use as many fingers as possible to bring a higher recognition and success rate. The experimental results show that with five fingers combined with area characteristic can reach a 95% successful login rate (Figure 9). Another finding is that the rotational velocity may change according to how familiar the user is with pbLogon, so it may not be useful as expected to help recognize users.

### 4.3. FAR and FRR Analyses

**Figure 10:**
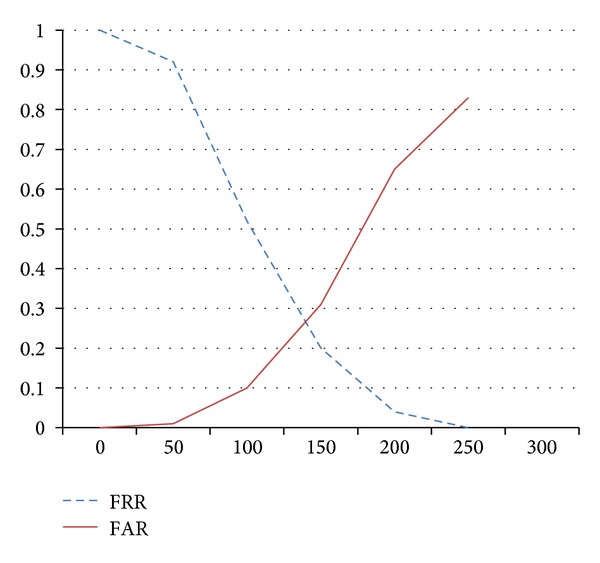
The FRR and FAR diagrams.

The performance of user authentication system may involve different criteria that sometimes it is more important to consider the false reject rate (FRR) and false acceptance rate (FAR).  Figure 10 shows the FAR and EER trends. The *x*-axis represents the different variance as thresholds, and *y*-axis is the corresponding rates. While the threshold setup is over 100 pixels, the false acceptance rate will increase up to 10%, which may be the barrier if the supervisor needs relatively high security environment. The FRR may also reveal the potential problems that users may need to perform more times to log into the system if the improper threshold is chosen. The equal error rate (EER) is about 26.8%, which means the performance may not be good as expected. However, it depends on usage of different scenarios and limitations.

### 4.4. Analysis Using* k*-Nearest Neighbors Algorithm

**Figure 11:**
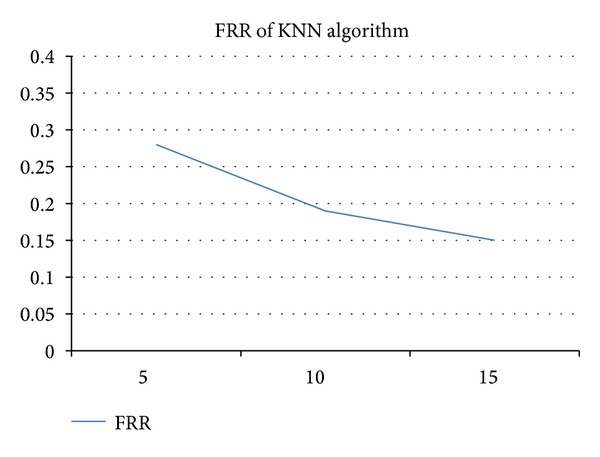
The experimental analysis using KNN algorithm.

The *k*-nearest neighbors algorithm is also adopted to evaluate the physiological biometrics. By the suggestion of SPSS, 80% of the user logs were used for training and 20% for prediction.  Figure 11 shows the analytic results using KNN module provided by SPSS. The *x*-axis is the number of records adopted for KNN analysis, and *y*-axis is the FRR. The result shows that the more the user operations are, the lower the FRR will be. And ten records can reduce the FRR to 20%, which is also similar to the experimental results of pbLogon. However, it is more complex and requires more computation power to perform KNN analysis. pbLogon has the benefit of quick response and portable to handheld devices for power saving propose.

### 4.5. Other Factors May Influence the Accuracy of pbLogon

Several additional factors influence the effectiveness of pbLogon biometrics: (1) the human hand is flexible object and the projection of its finger may suffer nonlinear deformations when multiple finger positions are acquired from the same person. That is especially true when users are untrained or noncooperative or are fooling the system. Improper thumb placement and little fingers that would not straighten were found by [41] to generate statically significant differences in matching scores. Mobile devices of different touch screen size may also suffer the similar problem. To handle this kind of problem, in the proposed scheme, we can slightly resize the images of virtual wheel lock to intimate the user to expand or narrow their fingers since we train and recognize by the relative position of the fingers. The natural pose restoration algorithm is also provided to extract the reliable hand pose for both training and verification purposes. (2) The sweating hand and emotional states will not affect the verification of pbLogon since it provides both physiological and behavioral biometric extractions. pbLogon also controls the user behavior by providing a fixed touching area and rotational speed. Therefore, if the user is not willing to login with the provided touching will be considered misuse and attacks. (3) The environment requirement of pbLogon system is only a multitouch device. Since only the touch coordinates and fingers' distance are required to perform user authentication, pbLogon has the ability to prevent other environmental facts, such as lights, temperatures, and other biases.

Chen et al. [42] showed that hand shape systems are vulnerable to spoof attacks. They build fake hands out of silhouette images captured by a HandKey II hand geometry reader and hand them to be accepted by the system. In the proposed scheme, we introduce the behavioral biometrics, which were implemented by using rotation dynamics which is alternatively hard to imitate.

### 4.6. Privacy and Replacement Issue

It is commonly known that the biometric trait of a person cannot be easily replaced. Once a biometrics is ever compromised, it would mean the loss of a user's identity forever. Therefore, protecting the biometric templates is a major concern and also a challenging task [43]. Cancellable biometrics is a way in which the biometric template is secured by incorporating the protection and replacement features into biometrics. A good cancellable biometrics formulation must fulfill four requirements: (1) diversity: the same cancellable template cannot be employed in two different applications; (2) reusability: straightforward revocation and reissue in the occurrence of compromise; (3) one-way permutation: implement nonreversible template calculation to avoid recovery of biometric data; (4) performance: the recognition performance should not be deteriorated by the formula.

**Figure 12:**
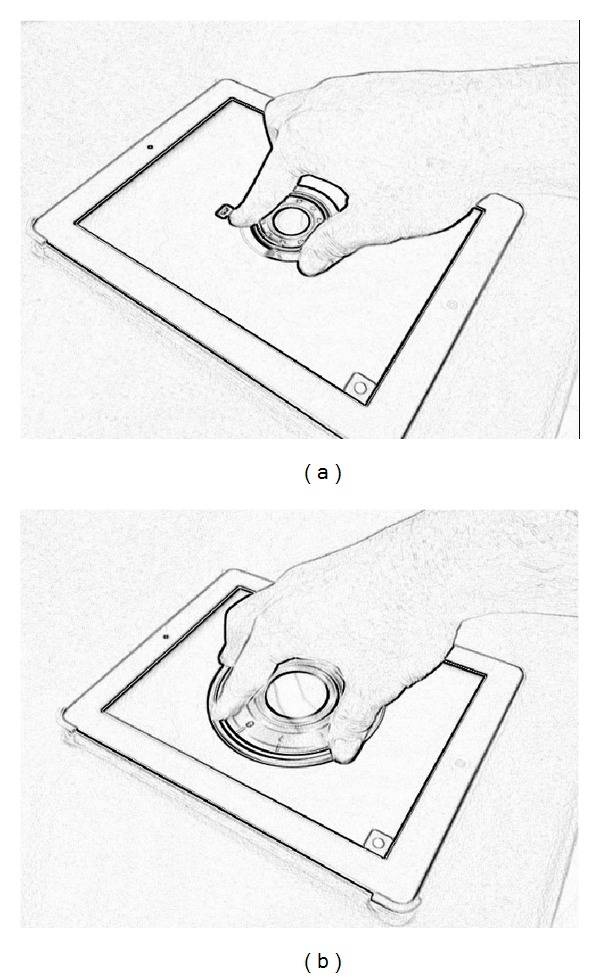
Example of different identities of the same user.

In the proposed scheme, the relative distance of different fingers was adopted to prevent privacy leakage. The replacement of users' identities can be easily achieved by changing the size and the type of carrier for verification.  Figure 12 demonstrates an example for changing the biometrics by resizing the wheel lock. The other ways to replace the existing users' biometric traits is to change the rotation speed of lock wheel. Dynamically adjusting the rotation speed of wheel lock can also affect the biometrics significantly, including the angles and other rotational dynamics. In addition, we can use Bayesian classifiers and neural network as the learning method; the requirements of one-way permutation can also be realized.

## 5. Conclusion

This study proposes a novel authentication approach consisting of both physiological and behavioral biometrics. The proposed scheme derives the possibility of performing complicated biometrics without extra equipment, but only multitouch panel integrated in most mobile devices. The experiments showed that it can be used to handle the general user authentication scenario and provide a relatively secure environment to prevent attacks. We also demonstrate how the biometric privacy can be obtained through the biometric identity replacement. The future works can be divided into experiment and implementation. The experiments will be used to verify and evaluate the feasibility of the proposed scheme with large-scale participants. With regard to implementation, multiple mobile devices, which have touch panel as interface, should be applicable or portable with the corresponding pbLogon in all respects.

## Figures and Tables

**Algorithm 1:**
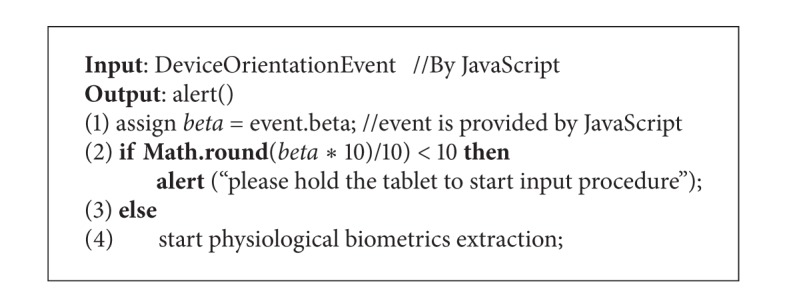
Check orientation algorithm.

**Algorithm 2:**
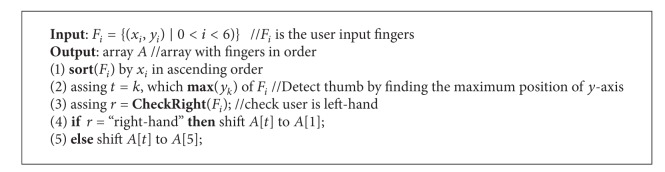
Hand natural pose restoration.

**Algorithm 3:**
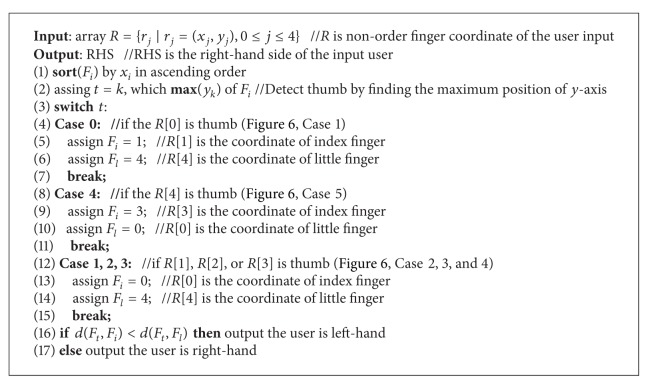
Check right-hand algorithm.

## Notations

MU: Mobile user MU = {*U*_*i*_∣*i* > 0}
*U*_*i*_: User profile of *i*th user
*P*: pbLogon system
*Password*: *n*-digits numeric password
*N*: *N* presents the total number of fingers on touch panel
*G*(·): *G*(·) returns the number of fingers, hand pose, and other biometric characteristics
*H*_*i*_: The *i*th hand of *U*_*i*_
*F*_*i*_: *i*th finger; ex: thumb presents as *F*_1_
*D*: *D* = {*d*_*ij*_∣0 < *i* ≠ *j* < 6}, where *d*_*ij*_ is the distance between fingers
*A*: *A* = {*a*_*ij**k*_∣0 < *i* ≠ *j* ≠ *k* < 6}, where *a*_*ij**k*_ is the area of any three fingers
*L*: *L* = {0,1∣*L* = 1 if *F*_2_ (index finger) is longer than *F*_4_ (ring finger), otherwise *L* = 0}
*R*: *R* = {0,1∣*R* = 0 if the user is left-handed, otherwise *R* = 1}
*V*: The set of the rotation velocity of the user; clockwise presents with positive value and counterclockwise presents in negative value
*B*_*i*_: Behavioral biometrics *B*_*i*_ of *U*_*i*_
*C*_*k*_(·): Classifiers with *k*th feature
LB_*ik*_: The low-bounds value of *k*th feature
UB_*ik*_: The up-bounds value of *k*th feature
FEA_*i*_: FEA_*i*_ = (LB_*ik*_, UB_*ik*_) of every feature can be directly obtained from the low-bounds and up-bounds of training patterns
Auth(·): The user authentication function
Δ*S*: The dissimilarity score
*Q* → *T* : *M*: Entity *T* receives a message *M* from *Q*.
